# Toxicity of synthetic cannabinoids is increasing along with the regulatory measures taken for their control

**DOI:** 10.1080/07853890.2021.1897430

**Published:** 2021-09-28

**Authors:** Joana Couceiro, Ana Luzio, Carla Ferreira, Alexandre Quintas

**Affiliations:** a Molecular Pathology & Forensic Biochemistry Laboratory, Centro de Investigação Interdisciplinar Egas Moniz (CiiEM), Egas Moniz Cooperativa de Ensino Superior, Caparica, Portugal; b Laboratório de Ciências Forenses e Psicológicas Egas Moniz, Centro de Investigação Interdisciplinar Egas Moniz (CiiEM), Egas Moniz Cooperativa de Ensino Superior, Caparica, Portugal

## Abstract

**Introduction:**

Novel psychoactive substances (NPS) represent a rising public health threat due to the associated side effects and the lack of success on their control policies. NPS are freely sold, mostly on the internet, as legal and safe replacements of controlled drugs of abuse. Synthetic cannabinoids (SCs) represent one of the largest groups of NPS and are designed as mimetics of Δ9-tetrahydrocannabinol from cannabis [1]. After the identification of JWH-018, the first SC appearing on the market, many countries took measures to control the free circulation of these products [2]. Nevertheless, efforts to control them are rapidly contoured, since clandestine manufacturers are constantly changing their chemical structures leading to previously unknown SCs [3]. The aim of this study is to show how chemical changes on recent generations of SCs to evade the law lead to the production of more harmful compounds, with potential application in forensic context. With that purpose, the toxicity of three SCs, representatives of the first, second and third generations [4], was assessed.

**Materials and methods:**

The SC JWH-018 was obtained as >98.5% pure powders from Lipomed AG Switzerland. SCs THJ-018 and EG-018 were bought through an internet website (www.chem.eu). THJ-018 and EG-018 were purified through HPLC/DAD and confirmed by GC/MS. Pure SCs stock solutions were prepared in 100% DMSO accordingly to the stipulated final concentrations and diluted into the culture medium prior addition to cells. The neuroblastoma human cell line SH-SY5Y was exposed, for 24 h, to several concentrations of each SC. Cell toxicity was evaluated through MTT assays.

**Results:**

Figure 1 shows the MTT results for SH-SY5Y viability in the presence of JWH-018, THJ-018 and EG-018. SH-SY5Y cells viability does not decrease in the presence of JWH-018 at the range of concentrations used. However, when the same cell line is exposed to THJ-018 or EG-018 there is a decrease in cell viability with the increase in the concentration of these substances. **Discussion and conclusions:** The results from the present study points THJ-018 and EG-018, JWH-018 analogues of 2nd and 3rd generations, as more toxic to the neuroblastoma cells than JWH-018, the first SC found on the street market. These results suggest that emerging modified SCs, to avoid the law, are becoming more toxic and dangerous. Given the emergence of this situation, it is time to rethink current legislation to prevent rise on public health issues derived from consumption of molecules of unknown toxicological profile.

**Figure 1.**
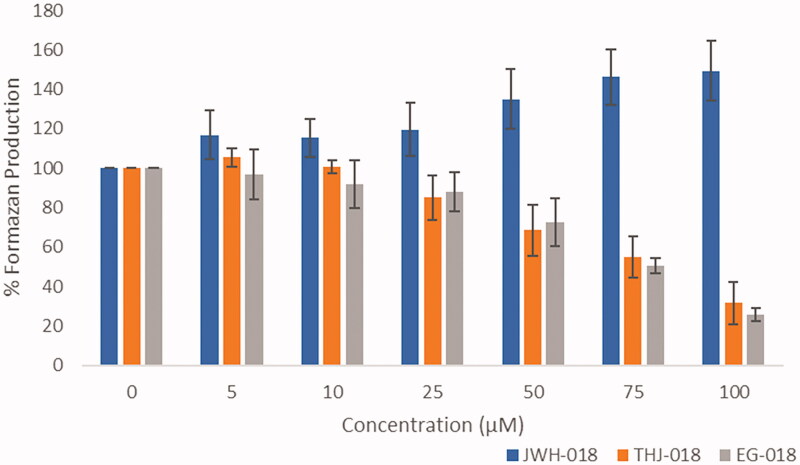
Cytotoxic evaluation by MTT assay of JWH-018, THJ-018 and EG-018 in SH-SY5Y cells. Data are expressed as the mean ± SD (*n* = 3).
